# Supplementary material for: Pilot Feasibility Assessment of a Tailored Physical Activity Prescription in Overweight and Obese People in a Public Hospital
Source: Int J Environ Res Public Health. 2022 Aug 30;19(17):10774. doi: 10.3390/ijerph191710774 (PMC9517763; doi:10.3390/ijerph191710774)
Supplement: Supplementary file 1 [file ijerph-19-10774-s001.zip › Table S1.pdf]

Table S1. Phases for physical activity prescription

Phase	Frequency and Duration	Description
Warming-up	5 – 10 minutes	Sequence of movements to activate the parts of the body to be exercised
Conditioning	30 minutes of **moderate intensity exercise** x 5 days/week<br>20 minutes of **vigorous intensity exercise** x 3 days/week<br>30 minutes of **moderate intensity exercise** x 2 days/week + 20 minutes of **vigorous intensity exercise** x 2 days/week	Includes aerobic exercises to improve cardiorespiratory function, and muscular strength exercises to improve muscular function
Cooling	5 – 10 minutes	Low-intensity exercise to restore heart function and blood pressure
Stretching	5 – 10 minutes	Exercises to improve muscle and tendon range of motion, postural stability and balance
