# Supplementary material for: Pilot Feasibility Assessment of a Tailored Physical Activity Prescription in Overweight and Obese People in a Public Hospital
Source: Int J Environ Res Public Health. 2022 Aug 30;19(17):10774. doi: 10.3390/ijerph191710774 (PMC9517763; doi:10.3390/ijerph191710774)
Supplement: Supplementary file 1 [file ijerph-19-10774-s001.zip › Table S2.pdf]

Table S2. Primary and secondary clinical outcomes at baseline, sixth and twelfth weeks

Outcome measures	Baseline (n=30)		Follow-up 6th week (n=11)		Final evaluation 12th week (n=21)	
**Primary**						
Physical Activity (MET-min/week) [1]						
Walking	1386	(2541.0)	1097.3	(1270.5)	2079	(3234)
Moderate Intensity	1305	(2430.0)	900	(1140.0)	2520	(3520)
Vigorous Intensity	0	0	1440	(2400)	0	(960)
Sitting time (hours/day) [1]	3	(3)	4	(2)	3	(3.3)
Six minutes' walk test [1]						
BR diff	4	(4)	4	(2)	4	(4)
HR diff	21	(24)	26.5	(21)	3	(14)
SBP diff	10	(13)	8	(12)	10	(10)
DBP diff	0	(12)	2	(10)	0	(4)
Borg Scale diff [1]						
Dyspnea	2	(3)	2	(2)	4	(4)
Fatigue	2	(3)	2	(3)	4	(4)
Walk Distance (m)	378.9	(59.8)	358.8	(52.4)	329.9	(87.4)
**Secondary**						
Body weight (Kg)	85.1	(12.9)	81.3	(12.3)	86.2	(13.0)
BMI (Kg/cm2)	33.4	(3.4)	33.6	(3.0)	33.9	(3.1)
Waist circumference (cm)	104.2	(9.7)	100.5	(9.8)	103.5	(10.1)
Heart rate	74.7	(9.5)	74	(7.8)	75.9	(8.7)
Blood pressure (mmHg)						
SBP	108.1	(8.9)	105.8	(8.6)	110.2	(7.4)
DBP	65.9	(8.2)	61.9	(10.5)	71.2	(7.4)
Quality of Life						
Physical fitness	3	(1.0)	2.6	(0.7)	2.9	(0.9)
Feelings	2.7	(1.4)	3.2	(1.3)	3	(1.3)
Daily activities	1.7	(0.9)	1.8	(1.2)	2	(1.1)
Social activities	1.5	(1.0)	2.1	(1.7)	2	(1.4)
Change in health	2.7	(0.8)	2.4	(0.8)	2.7	(1.2)
Overall health	3.6	(0.8)	3.4	(1.0)	3.3	(1.1)

Note: DBP, diastolic blood pressure (mmHg); SBP, systolic blood pressure (mmHg); BR, breathing rate (breaths per minute), HR, heart rate (beats per minute)

[1] Medians and interquartile range are shown
