# Supplementary material for: Pilot Feasibility Assessment of a Tailored Physical Activity Prescription in Overweight and Obese People in a Public Hospital
Source: Int J Environ Res Public Health. 2022 Aug 30;19(17):10774. doi: 10.3390/ijerph191710774 (PMC9517763; doi:10.3390/ijerph191710774)
Supplement: Supplementary file 1 [file ijerph-19-10774-s001.zip › Table S3.pdf]

Table S3. Changes in primary and secondary clinical outcomes sixth and twelfth weeks compared with the baseline in a crude model

Outcome measure	Crude Model					
	Follow-up (6th week)			Final evaluation (12th week)		
	Coef. (IC 95%)		p-value*	Coef. (IC 95%)		p-value [1]
**Primary**						
Physical Activity (MET-min/week)						
Walking	-505.2	(-1566.3 - 556.0)	0.351	813.2	(-137.0 - 1763.5)	0.093
Moderate Intensity	-619.1	(-1491.3 - 253.1)	0.164	892.3	(-10.2 - 1794.9)	0.053
Vigorous Intensity	2290.7	(278.5 - 4302.9)	0.026	790.8	(-250.8 - 1832.5)	0.137
Sitting time (hours/day)	-0.2	(-1.2 - 0.76)	0.664	0.1	(-1.4 -1.7)	0.877
Six minutes' walk test						
BR diff	-1.6	(-3.1 - -0.2)	0.029	-1.1	(-3.1 - 0.8)	0.253
HR diff	-6.4	(-15.5 - 2.7)	0.17	-21.4	(-29.9 - -12.9)	0.001
SBP diff	-2.6	(-7.9 - 2.7)	0.332	-0.4	(-7.0 - 6.1)	0.898
DBP diff	1.9	(-3.9 - 7.7)	0.527	-3.9	(-8.4 - 0.6)	0.092
Borg Scale diff						
Dyspnea	-0.5	(-1.8 - 0.8)	0.46	1.3	(-0.8 - 3.4)	0.228
Fatigue	0.1	(-1.6 - 1.9)	0.877	1.5	(-0.2 - 3.3)	0.084
Walk Distance (m)	-19.5	(-57.8 - 18.9)	0.319	-48.4	(-90.5 - -6.4)	0.024
**Secondary**						
Body weight (Kg)	0.2	(-0.6 - 1.1)	0.572	-0.2	(-1.4 - 0.9)	0.689
BMI (Kg/cm2)	0.1	(-0.2 - 0.5)	0.396	-0.1	(-0.6 - 0.5)	0.699
Waist circumference (cm)	-1.1	(-2.8 - 0.6)	0.205	-1.6	(-3.2 - 0.03)	0.055
Heart rate	0.9	(-4.1 - 6.0)	0.713	0.4	(-3.4 - 4.3)	0.817
Blood pressure (mmHg)						
SBP	-0.6	(-4.8 - 3.5)	0.761	0.5	(-3.0 - 4.0)	0.760
DBP	-3.9	(-10.4 - 2.5)	0.234	5.0	(1.6 - 8.4)	0.004
Quality of Life						
Physical fitness	-0.2	(-0.5 - 0.4)	0.335	-0.04	(-0.5 - 0.4)	0.859
Feelings	0.6	(0.03 - 1.2)	0.04	0.3	(-0.3 - 0.9)	0.339
Daily activities	0.1	(-0.5 - 0.7)	0.735	0.3	(-0.2 - 0.9)	0.206
Social activities	0.6	(-0.1 - 1.3)	0.114	0.4	(-0.2 - 0.9)	0.212
Change in health	-0.5	(-1.0 - 0.6)	0.081	0.1	(-0.5 - 0.6)	0.858
Overall health	-0.2	(-0.8 - 0.3)	0.446	-0.3	(-0.8 - 0.2)	0.209

[1] p-value = 0.025 using Bonferroni correction
